# Protection from Experimental Cerebral Malaria with a Single Dose of Radiation-Attenuated, Blood-Stage *Plasmodium berghei* Parasites

**DOI:** 10.1371/journal.pone.0024398

**Published:** 2011-09-15

**Authors:** Noel J. Gerald, Victoria Majam, Babita Mahajan, Yukiko Kozakai, Sanjai Kumar

**Affiliations:** Division of Emerging Transfusion Transmitted Diseases, Center for Biologics Evaluation and Research, Food and Drug Administration, Rockville, Maryland, United States of America; State University of Campinas, Brazil

## Abstract

**Background:**

Whole malaria parasites are highly effective in inducing immunity against malaria. Due to the limited success of subunit based vaccines in clinical studies, there has been a renewed interest in whole parasite-based malaria vaccines. Apart from attenuated sporozoites, there have also been efforts to use live asexual stage parasites as vaccine immunogens.

**Methodology and Results:**

We used radiation exposure to attenuate the highly virulent asexual blood stages of the murine malaria parasite *P. berghei* to a non-replicable, avirulent form. We tested the ability of the attenuated blood stage parasites to induce immunity to parasitemia and the symptoms of severe malaria disease. Depending on the mouse genetic background, a single high dose immunization without adjuvant protected mice from parasitemia and severe disease (CD1 mice) or from experimental cerebral malaria (ECM) (C57BL/6 mice). A low dose immunization did not protect against parasitemia or severe disease in either model after one or two immunizations. The protection from ECM was associated with a parasite specific antibody response and also with a lower level of splenic parasite-specific IFN-γ production, which is a mediator of ECM pathology in C57BL/6 mice. Surprisingly, there was no difference in the sequestration of CD8+ T cells and CD45+ CD11b+ macrophages in the brains of immunized, ECM-protected mice.

**Conclusions:**

This report further demonstrates the effectiveness of a whole parasite blood-stage vaccine in inducing immunity to malaria and explicitly demonstrates its effectiveness against ECM, the most pathogenic consequence of malaria infection. This experimental model will be important to explore the formulation of whole parasite blood-stage vaccines against malaria and to investigate the immune mechanisms that mediate protection against parasitemia and cerebral malaria.

## Introduction

Studies of natural immunity to human malaria and evidence from experimental models suggest that repeated exposure to malaria parasites is the most effective method to induce immunity against malaria [1]. Likewise, the most successful experimental malaria vaccine is based on repeated immunizations with radiation-attenuated malaria sporozoites delivered by multiple bites from infected mosquitoes [2]. In *Plasmodium falciparum* malaria, the immunity induced by irradiated sporozoites is species-dependent yet it is cross-protective against different parasite strains [3]. Attenuated parasite vaccines have long been an interest for malaria [1], and so far the major efforts to develop such whole organism vaccines have focused on generating attenuated sporozoites by radiation [4], [5], chemical [6], drug cure [7], or targeted gene disruption methods [8], [9], [10]. In comparison, less research has been done on live vaccines against the malaria blood stages which are responsible for the clinical symptoms of the disease [1], [11]. However, attenuated blood-stage vaccines produced by radiation [12], [13] gene disruption [14], [15], [16], and drug cure methods [17], [18], have demonstrated effectiveness for protection against parasitemia and symptoms of severe malaria.

In the current study, we investigate the effectiveness of a radiation-attenuated blood-stage parasites for protection against parasitemia and severe disease in experimental models of malaria. We used the highly virulent murine malaria parasite *Plasmodium berghei ANKA* (*Pb-A*) which, depending on the mouse genetic background, produces two distinct yet uniformly fatal pathologies. In CD1 mice, a few virulent blood-stage *Pb-A* are sufficient to initiate a patent infection that ultimately produces high parasite parasitemia, severe anemia, and death. In contrast, C57BL/6 mice infected with *Pb-A* are susceptible to experimental cerebral malaria (ECM) which is characterized by an early onset of neurological defects, coma, and death associated with a relatively low parasitemia [19], [20]. We show that a single, non-adjuvanted immunization with a high-dose of radiation-attenuated, blood-stage *Pb-A* parasites protected CD1 mice from parasitemia and severe disease, and it protected C57BL/6 mice from ECM. Protection from ECM was associated with an anti-parasite antibody response and a reduced IFN-γ response in the spleen during a virulent infection.

## Materials and Methods

### Mice, parasites, and immunizations

#### Ethics statement

6–8 week old female C57BL/6 and Swiss-CD1 mice were used in accordance with the animal study protocols (#2009-22, 2007-14) approved by the Food and Drug Administration, Center for Biologics Evaluation and Research Institutional Animal Care and Use Committee. Blood-stage *Plasmodium berghei* ANKA (*Pb-A*) parasites (an uncloned parasite line) from −70 C glycerolyte stocks were injected intraperitoneally into donor mice with the same genetic background of the experimental mice [19]. Parasitemia was monitored by Giemsa-stained thin blood smears and total RBC were counted by hemocytometer. For challenge infections, 10^4^ parasitized RBC (pRBC) were injected intravenously (iv) via the tail vein. Mice were evaluated for ECM symptoms and the ability to survive beyond day 10 post infection as previously described [20]. For immunization, 10^3^ or 10^7^ irradiated pRBC were injected into mice iv as described in the text.

### Radiation-attenuation of parasite growth

To attenuate parasite growth, freshly harvested *Pb-A* pRBC were diluted in phosphate buffered saline (PBS) to a concentration of 5×10^7^ pRBC per milliliter (ml). One ml aliquots of parasites were then immediately exposed to a Cesium-137 source for various time periods at room temperature in a Gammacell 1000 irradiator. Radiation dose was calculated from the machine-specific estimate of 1505 Rads per minute.

### Spleen cell culture

Culture media and buffers were obtained from Invitrogen unless specified otherwise. Freshly isolated mouse splenocytes were plated in triplicate in 24-well tissue culture plates. For parasite antigen stimulation, pRBC were lysed with 4 freeze/thaw cycles, and 1×10^6^ pRBC equivalents were added per well. Control wells were stimulated with an equal number of lysed uninfected RBC, or with medium alone. Cells were cultured for 72 hours, then supernatants were collected by centrifugation and stored at −70 C. Interferon-gamma protein levels were assayed in culture supernatants using the Ready-Set-Go sandwich ELISA kit (Ebioscience) according to the manufacturer's instructions with a stated detection sensitivity limit of 15 pg/ml.

### Brain sequestered cells

Brain cell suspensions were prepared as described [21]. Briefly, the brains of anesthetized mice were perfused intracardially with Hank's Buffered Saline Solution (HBSS), removed, and then pushed through 70 micron filters. Cell suspensions were centrifuged at 400× g at 21 C, and the pellets were resuspended in RPMI/FCS with 0.5 mg/ml collagenase D (Roche), 3 units/ml DNAse (Roche), and 5 mM MgCl_2_. The samples were rotated for 60 minutes at room temperature, allowed to stand for 10–15 minutes, and the supernatants were collected. Each sample was brought to a final concentration 33% Percoll (Sigma-Aldrich) and then under-layered with 70% Percoll/HBSS. The gradients were centrifuged at 515× g for 30 minutes at 21 C, the cells were collected from the 33/70% interface, and washed with HBSS. Residual red blood cells were lysed with ACK lysis buffer, and the samples were washed twice in RPMI/FCS before preparation for multicolor flow cytometry.

### Flow Cytometry

Brain cells (pooled from 4 mice within each group) were stained with the Fixable Viability Dye eFluor 660 (eBioscience) according to the manufacturer's instructions. Cells were blocked with TruStain fcX anti-mouse CD16/32 (Biolegend) and then stained in HBSS, 1% BSA on ice for 30 minutes with the following anti-mouse antibodies: AlexaFluor 488-CD8a (Biolegend), PE-NK1.1 (Biolegend), PerCP-CD11b (Biolegend), APC-eFluor 780-CD45 (eBioscience), eFluor 450-CD3, AlexaFluor488-CD44 (Biolegend), PE-CD62L (Biolegend), PerCP-CD69 (Biolegend), APC-eFluor 780-CD8a (eBioscience), and then fixed with IC fixation buffer (eBioscience). Samples were read on an LSRII cytometer (BD). Fluorescence-minus-one (FMO) controls were used to set population gates for each panel. Data analysis was performed in FlowJo v7.5 software. 36,000–160,000 cells were analyzed for each pooled brain sample.

### Serum antibody ELISA

Mouse sera were pooled within treatment groups and then stored at −20 C. *Pb-A*-specific antibodies were detected by endpoint ELISA at O.D. 405 nm as previously described [22]. Positive signal cut-off was defined as two times the mean O.D. value from normal sera. Antibody titer was determined as the highest sample dilution which produced an O.D. value greater than or equal to the cut-off.

### Statistics

Graphs, survival analysis, and statistics were performed in GraphPad Prism 5 (Graphpad Software, Inc). Two-sided p-values and 95% confidence intervals are reported. Survival to day 10 in ECM experiments was analyzed using Fisher's exact test as previously described [20]. For Two-Way ANOVA, the Bonferroni correction for multiple comparisons on post tests was used.

## Results

### Determination of the radiation dose necessary to attenuate blood-stage *Pb-A*

**Figure 1:**
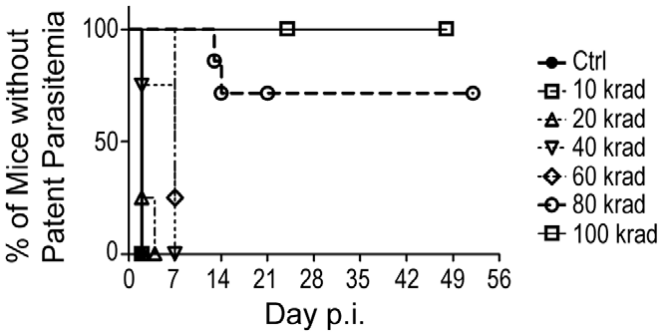
Determination of the radiation dose necessary to attenuate blood-stage *Pb-A* parasites. Virulent *Pb-A* parasitized RBC (pRBC) were exposed to increasing doses of gamma irradiation (10, 20, 40, 60, 80, 100 kilorads). 10^7^ irradiated pRBC or non-irradiated control pRBC (Ctrl) were injected intravenously (iv) into groups of naïve CD1 mice and parasitemia was monitored by blood smear. The graph shows the percentage of mice without patent parasitemia in each group over time beginning with day 2. Data are pooled from 3 experiments. Groups of mice infected with control pRBC or with pRBC exposed to 10, 20, 40, or 60 krad irradiation all developed patent parasitemia by day 7 (n = 4 mice each group). Accordingly, the curves describing these groups approach 0% by day 7. 70% (10/14) of mice infected with 80 krad irradiated pRBC remained free from patent blood-stage disease by day 14 (open circles, dashed line). 100% (10/10) of the mice injected with 100 krad irradiated pRBC remained free from blood stage infection under the period of observation (open squares, solid line). 100 krad irradiation was chosen as the attenuating dose in subsequent experiments.

To determine the radiation dose necessary to attenuate *Pb-A* growth and virulence, parasitized red blood cells (pRBC) were exposed to increasing doses of radiation. Parasite attenuation was evaluated by intravenously (iv) injecting 10^7^ irradiated pRBC into CD1 mice and then monitoring the mice for parasite growth and disease symptoms (Figure 1). Parasites irradiated at doses up to 60 kilorads reproduced in the mice, and these mice developed patent parasitemias within 7 days (Figure 1, Ctrl, 10–60 krad groups). Higher radiation doses produced greater evidence of parasite attenuation. All of the mice that received 80 kilorad irradiated parasites (14/14) were free of patent infection through day 7, and 70% (10/14) did not develop detectable parasitemias or disease symptoms for the duration of the experiment (Figure 1, open circles). However, by day 14, 30% (4/14) of the mice that had been injected with 80 kilorad irradiated parasites developed patent infections and severe disease symptoms. This suggested that the 80 kilorad irradiation attenuated the growth and virulence of the majority of the parasites, but that a few residual parasites escaped attenuation and were eventually able to establish fulminant infections.

In contrast, all of the mice (10/10) that received 100 kilorad irradiated parasites (Figure 1, open squares, solid line) remained free from patent parasitemia and disease symptoms for the duration of the experiment. This demonstrated that the 100 kilorad dose was sufficient to cause the complete attenuation of blood-stage *Pb-A* parasites, and so the 100 kilorad dose was chosen to attenuate parasite growth in subsequent experiments.

### Attenuated *Pb-A* blood-stage parasites induce protection against parasitemia and severe disease in CD1 mice

**Figure 2:**
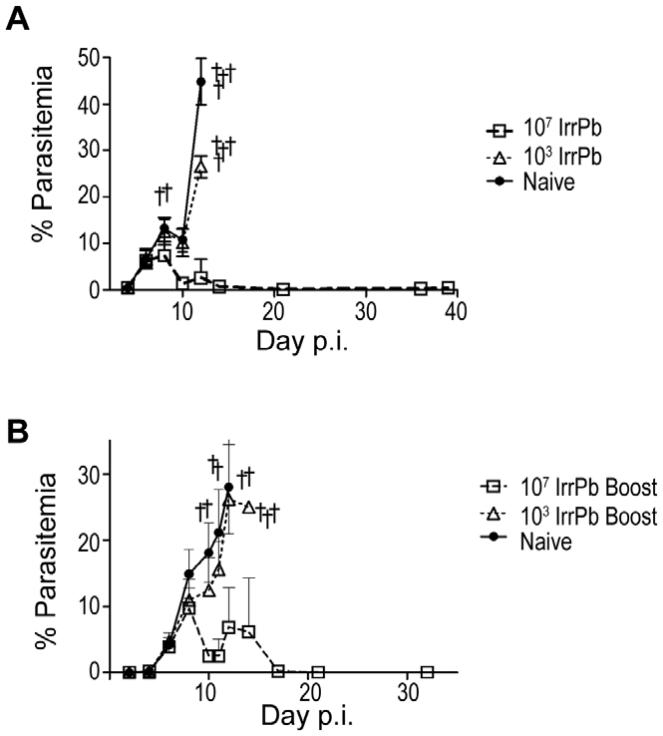
Protection from parasitemia and severe disease in CD1 mice after immunization with irradiated blood-stage *Pb-A*. **A**) Protection in CD1 mice after a single immunization with 10^7^ irradiation attenuated *Pb-A* blood-stage parasites (IrrPb). 10^3^ or 10^7^ IrrPb were injected iv into groups mice. On day 24 after injection, immunized and naïve mice were challenged with 10^4^ virulent *Pb-A* pRBC and parasitemia was monitored by blood smear (mean +/− SD) Daggers indicate mice that were euthanized or that died during the experiment. All naïve mice (4/4, filled circles) and 10^3^ IrrPb immunized mice (5/5, open triangles) displayed uncontrolled parasite growth and succumbed to acute blood-stage infection by day 12 after challenge. In contrast, mice immunized with 10^7^ IrrPb (4/5, open squares) controlled parasite growth after challenge and managed blood parasitemia down to low or undetectable levels. **B**) Protection in CD1 mice after a two immunizations with 10^7^ IrrPb. Groups of mice were immunized with 10^3^ or 10^7^ IrrPb, and then given a boost immunization 24 days later with an equal number of IrrPb (Boost). 24 days after the final immunization, immunized and naïve mice were challenged with 10^4^ virulent *Pb-A* pRBC. In mice immunized with 2 doses of 10^3^ IrrPb (n = 5 mice, open triangles), parasitemia levels continued to increase after challenge, similar to naïve mice (n = 5 mice, filled circles). In contrast, when mice were immunized with 2 doses of 10^7^ IrrPb (n = 5 mice, open squares), blood parasitemia declined over time to low or undetectable levels.

We determined whether iv immunization with 10^3^ or 10^7^ irradiated *Pb-A* blood-stage parasites (IrrPb) protected CD1 mice against parasitemia and/or severe disease after a challenge infection with virulent blood-stage *Pb-A* (Figure 2A). No residual IrrPb were detected in the blood smears of immunized mice on the day before the virulent challenge. After challenge, the group immunized with 10^3^ IrrPb displayed an uncontrolled blood infection similar to naïve mice, and none of these mice (0/5) survived beyond day 12 without developing severe disease symptoms. In contrast, the group immunized with 10^7^ IrrPb (n = 5, Figure 2A, open squares) showed evidence of controlling the blood stage infection after challenge, and reached a peak mean parasitemia of 7.3% on day 8 with only one of these mice developing severe disease symptoms on this day. 80% (4/5) of the 10^7^ IrrPb immunized mice did not develop severe malaria symptoms and their mean parasitemias decreased to low or undetectable levels by day 21 (Figure 2A). These mice remained free from disease symptoms for 5 months of observation after challenge. This is strong evidence that a single immunization with 10^7^ IrrPb protects CD1 mice from virulent challenge, while a similar immunization with 10^3^ IrrPb is not protective.

To test whether more than one immunization of attenuated parasites would improve protection, groups of CD1 mice were immunized with 10^3^ or 10^7^ IrrPb and 24 days later they were given a booster immunization with an equal number of IrrPb. The mice were then challenged with virulent *Pb-A* pRBC and parasitemia was monitored (Figure 2B). Similar to naïve mice (Figure 2B, filled circles), mice immunized twice with 10^3^ IrrPb (Figure 2B, open triangles) displayed continually increasing parasite growth after challenge. Mice that had received two immunizations with 10^7^ IrrPb (n = 5, Figure 2B, open squares) developed a peak mean parasitemia of 9.7% on day 8 after challenge, and by day 21, all of these mice (5/5) had controlled parasitemias down to low or undetectable levels. Similar to the results from single immunization experiments (Figure 2A), these results demonstrated that two immunizations with 10^7^ IrrPb was protective in a model of acute blood-stage disease, while two immunizations with 10^3^ IrrPb was not protective.

### Attenuated *Pb-A* blood-stage parasites induce protection against ECM in C57BL/6 mice

**Figure 3:**
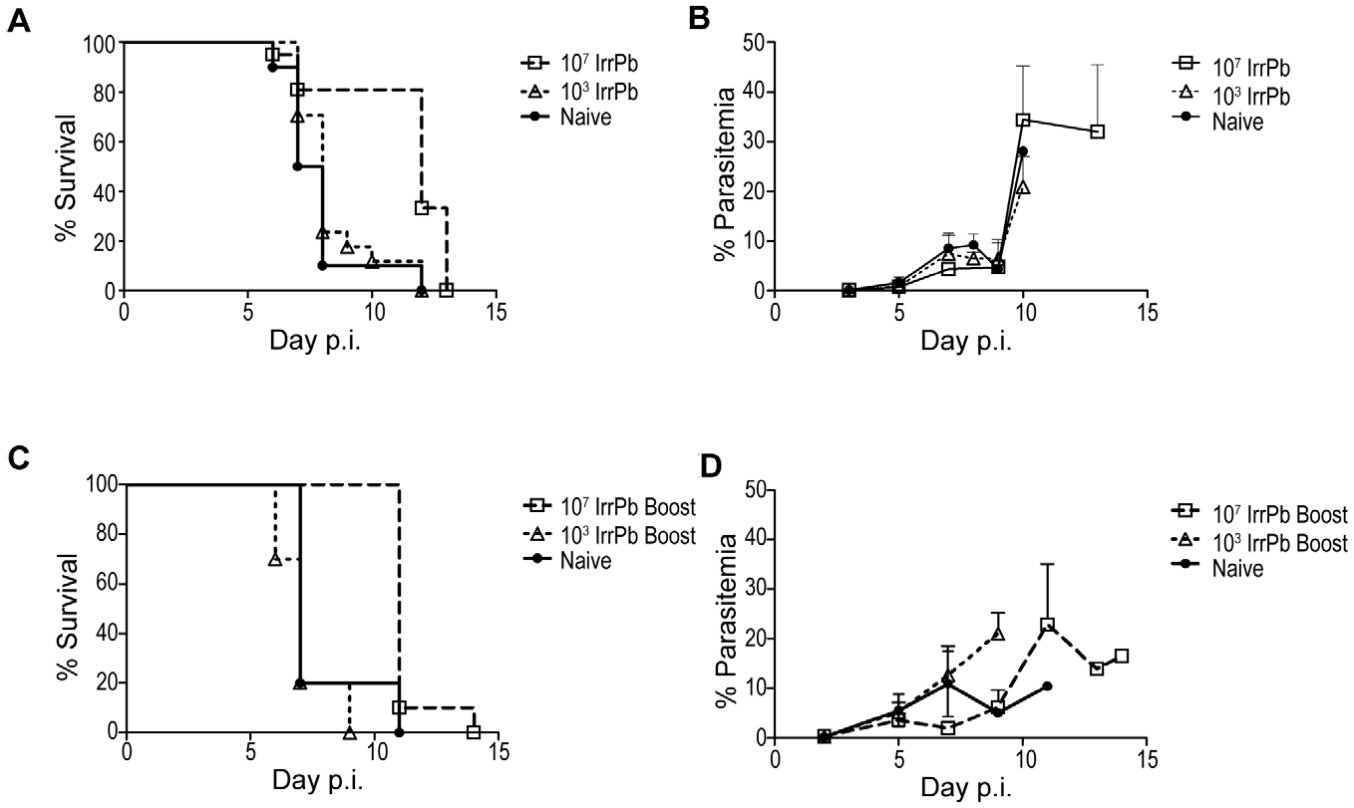
Protection from experimental cerebral malaria (ECM) in C57BL/6 mice after immunization with IrrPb. **A and B**) Protection from ECM after a single immunization of 10^7^ IrrPb. Data are pooled from two independent experiments. Groups of mice were immunized with 10^3^ or 10^7^ IrrPb and then challenged with 10^4^ virulent *Pb-A* pRBC on day 19 or 28 after immunization. To assess ECM, challenged mice were monitored for neurological symptoms, the ability to survive beyond day 10 (A), and blood parasitemia levels (B). **A**) 10% (1/10) of naïve mice (filled circles) and 12% (2/17) of mice immunized with 10^3^ IrrPb (open triangles) survived beyond day 10 after challenge. In contrast, 81% (17/21) of mice immunized with 10^7^ IrrPb (open squares) survived beyond day 10 (two-sided p = .0003, 10^7^ IrrPb group vs. naïve group, Fisher's exact test). **B**) In each group, mice that succumbed to ECM before day 10 had blood parasitemias less than 10%, while mice that survived beyond day 10 eventually developed hyperparasitemia. **C and D**) Protection from ECM after two immunizations with 10^7^ IrrPb. Mice were immunized with 10^3^ or 10^7^ IrrPb twice with a 28 day interval (Boost), challenged with 10^4^ virulent pRBC, and then monitored for survival (C) and blood parasitemia (D). **C**) 20% (1/5) of naïve mice (filled circles) and none (0/10) of the mice immunized twice with 10^3^ attenuated parasites (open triangles) survived beyond day 10 after challenge. In contrast, all (10/10) of the mice immunized with 10^7^ attenuated parasites (open squares) survived beyond day 10 (p = .0037, 10^7^ IrrPb group vs. naïve group, Fisher's exact test), and eventually developed hyperparasitemia (D).

Since immunization with IrrPb protected CD1 mice from parasitemia and acute blood-stage disease, we wanted to test whether a similar immunization regimen would protect C57BL/6 mice from the symptoms of experimental cerebral malaria (ECM). Groups of C57BL/6 mice immunized once with 10^3^ or 10^7^ IrrPb were challenged with virulent *Pb-A* pRBC and then monitored for the symptoms of ECM, survival beyond day 10, and blood parasitemia (Figures 3A and 3B). After challenge, 10% (1/10) of naïve mice (filled circles) and 12% (2/17) of mice immunized with 10^3^ IrrPb (open triangles) survived beyond day 10 (Figure 3A). The mean parasitemia levels were below 10% for all groups before day 10 (Figure 3B). This is consistent with the ECM model in C57BL/6 mice, and suggested that immunization with 10^3^ IrrPb did not offer any significant protection from ECM or parasitemia. In contrast, 81% (17/21) of mice immunized with 10^7^ IrrPb (open squares) did not display any symptoms of ECM and survived beyond day 10 (Figure 3A, p = .0003 survival to day 10, naïve vs. 10^7^ IrrPb immunized mice). This is very strong evidence that a single immunization with 10^7^ IrrPb induced a high level of protection from ECM. Similar to other reports in this model [23], the mice that were protected from ECM and survived beyond day 10 still succumbed to hyperparasitemia by the end of the experiment (Figure 3B).

To test whether two immunizations with IrrPb enhanced protection from ECM, C57BL/6 mice immunized twice with 10^3^ or 10^7^ IrrPb with a 28 day interval were challenged with virulent *Pb-A* pRBC (Figures 3C and 3D). After challenge, 20% (1/5) of naïve mice (filled circles) and none (0/10) of the mice immunized twice with 10^3^ IrrPb (open triangles) survived to day 10, indicating that two immunizations did not improve the ability of 10^3^ IrrPb to induce protection against ECM in this model. In contrast, 100% (10/10) of the mice immunized twice with 10^7^ IrrPb (open squares) survived to day 10 after challenge (p = .0037, survival to day 10, naïve vs. 10^7^ IrrPb boost immunized mice), which was strong evidence that two immunizations with 10^7^ IrrPb protected from ECM. Immunization with 10^7^ IrrPb did not protect against parasitemia in the C57BL/6 model since all mice that survived ECM eventually succumbed to high parasite levels (Figure 3B and D).

### Protective immune responses associated with attenuated *Pb-A* blood-stage parasites

**Figure 4:**
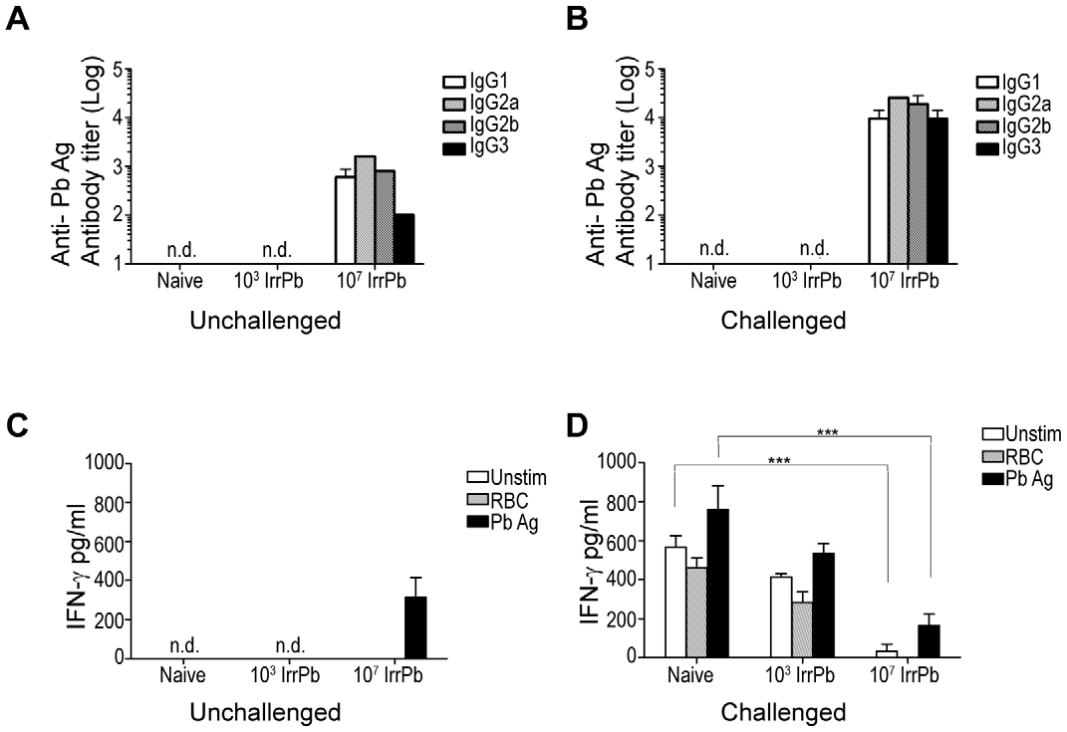
Immune responses associated with IrrPb immunization in C57BL/6 mice. C57BL/6 mice were immunized once with 10^3^ or 10^7^ IrrPb, challenged with virulent pRBC, and tissues were harvested on day 6 when ECM appeared in naïve mice. **A and B**) Serum IgG antibodies (subclasses IgG1, IgG2a, IgG2b, and IgG3) to blood-stage *Pb-A* antigen were measured by endpoint ELISA. Values are averaged from two independent experiments. Parasite-specific IgG antibodies were not detected (n.d.) in sera from naïve mice or 10^3^ IrrPb immunized mice before (A) or after challenge (B) with virulent pRBC. Parasite-specific IgG antibodies were detected in sera from 10^7^ IrrPb immunized mice before challenge (A), and the antibody titers increased between 1 to 2 log-fold in the sera of these mice after challenge (B). **C and D**) Cellular responses: IFN-γ responses in the spleen during a virulent infection are reduced in immunized mice. Spleen cells from unchallenged (C) or challenged (D) mice were cultured with blood-stage *Pb-A* antigen (Pb Ag), uninfected RBC (RBC), or media control (Unstim). IFN-γ protein was measured in cell culture supernatants by sandwich ELISA (mean +/− SEM) **C**) Among unchallenged mice, IFN-γ was not detected (n.d.) in samples from naïve mice or 10^3^ IrrPb immunized mice. A low level of IFN-γ was detected in samples from 10^7^ IrrPb immunized mice after stimulation with parasite antigen (Pb Ag, black bar). **D**) During a virulent infection, the highest levels of IFN-γ were detected in samples from naïve and 10^3^ IrrPb immunized mice. 10^7^ IrrPb immunized mice had lower levels of antigen-stimulated IFN-γ in comparison to antigen-stimulated samples from naïve mice (black bars, mean difference −596 (411, 781) pg/ml, p<.0001, Two Way ANOVA).

We next investigated the antibody and cellular responses induced by a single immunization with 10^3^ or 10^7^ IrrPb for correlations with the protection from ECM. *Pb-A*-specific IgG antibodies were not detected in sera from 10^3^ IrrPb immunized mice with or without challenge, similar to naïve mice (Figure 4A and B). In contrast, *Pb-A*-specific IgG antibodies were detected in sera from 10^7^ IrrPb immunized mice before challenge (Figure 4A), and antibody titers were detected at levels 1–2 log-fold higher in the sera from these mice during a virulent challenge (Figure 4B). Thus, the immunization with 10^7^ IrrPb which was protective against ECM generated a parasite-specific serum antibody response, while the non-protective immunization with 10^3^ IrrPb did not produce a measureable antibody response.

Parasite-specific IFN-γ responses were also measured in spleen cell cultures from immunized mice before or during a virulent infection (Figure 4C and 4D). Among uninfected mice, IFN-γ was not detected in samples from naïve or 10^3^ IrrPb immunized mice (Figure 4C, n.d). However, samples from uninfected 10^7^ IrrPb immunized mice did produce IFN-γ protein in response to *Pb* antigen stimulation (Figure 4C, black bar, PbAg), indicating that immunization with only the higher dose of IrrPb was able to induce IFN-γ production by spleen cells in the absence of a virulent infection.

During a virulent *Pb-A* challenge infection, the spleen cells from naïve mice and 10^3^ IrrPb immunized mice produced the highest levels of IFN-γ (Figure 4D). As described in Figure 3, these groups had the highest prevalence of ECM, and this pattern of IFN-γ expression is consistent with published reports. In contrast, during a virulent infection the spleen cells from the 10^7^ IrrPb immunized mice produced significantly less IFN-γ in comparison with cells from naïve mice, whether they were stimulated in vitro with parasite antigen (Figure 4D, black bars. p<.0001, naïve vs. 10^7^ IrrPb, mean difference 596 (411, 781) pg/ml) or unstimulated (Figure 4D, white bars. p<.0001, naïve vs. 10^7^ IrrPb, mean difference 534 (349, 719) pg/ml).

**Figure 5:**
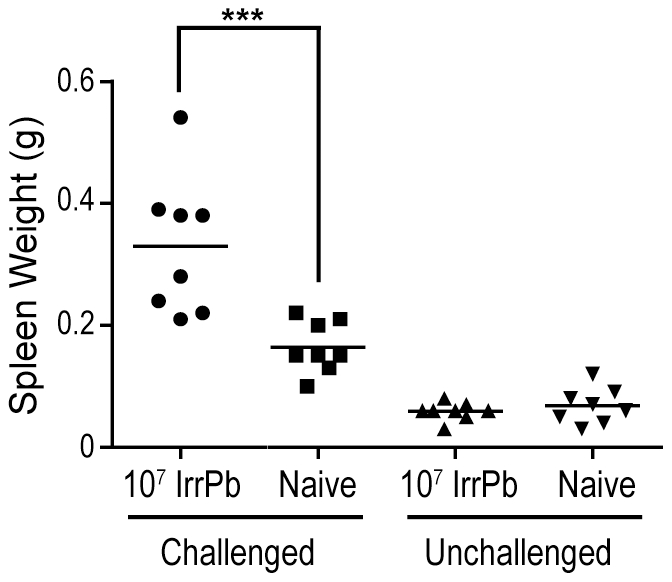
Spleen weights of mice immunized with IrrPb increase during a virulent infection. C57BL/6 mice were immunized once with 10^7^ IrrPb, challenged with virulent parasites, and then spleens were harvested on day 6 after challenge. Spleens from immunized mice after challenge were an average 0.17 grams (0.09, 0.24) larger than the spleens from naïve mice after challenge (two-sided p<.001, Two Way ANOVA, Bonferroni post test). Results (n = 8 mice in each group) are pooled from two independent experiments.

Since a single immunization with 10^7^ IrrPb 1) protected mice from ECM, 2) produced a parasite-specific antibody response, and 3) was associated with a decreased IFN-γ response in the spleen during infection, we decided to examine the response to this immunization further. Consistent with other studies using attenuated blood-stage *Pb-A*, the average spleen weights from immunized challenged mice (Figure 5, 10^7^ IrrPb Challenged) were 1.5–2-fold larger than the spleen weights from naïve challenged mice (p<.001, mean difference = 0.17 (0.09, 0.24) grams), suggesting an increased splenic activity during infection. In the absence of a virulent *Pb-A* challenge, the spleens from immunized mice were not significantly heavier than the spleens from naïve mice (Figure 5, Unchallenged).

**Figure 6:**
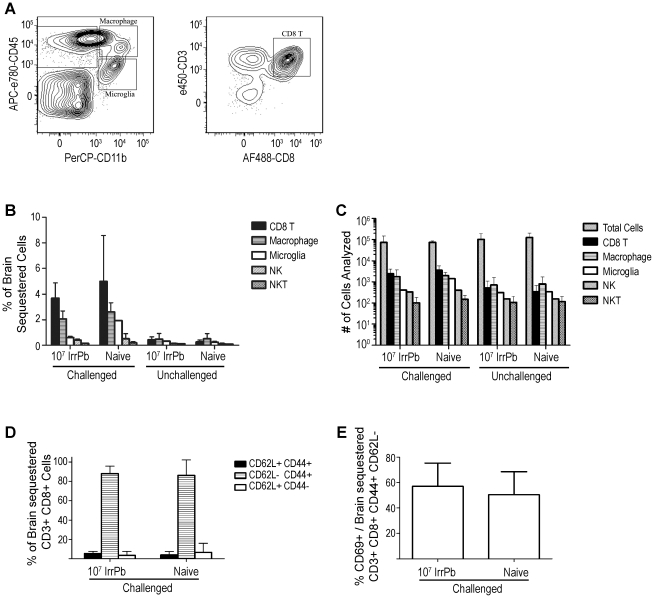
Flow cytometry analysis of cell subtypes in brains of mice immunized with 10^7^ IrrPb. Brain samples were processed on day 6 after virulent challenge and were pooled from all 4 mice in each group. Results are combined from two independent experiments. **A**) Representative cell plots from naïve mouse brain samples during a virulent infection. Gates for macrophages (CD45+, CD11b+), microglia (CD45int, CD11b+), and CD8 T cells (CD45+, CD3+, CD8+) are indicated. **B**) Graph of brain sequestered cell frequencies. Naïve mice had increased frequencies of brain-sequestered CD8 T cells and macrophages during a virulent challenge (Naïve Challenged, black and hatched bars). Immunized challenged mice displayed frequencies of brain-sequestered CD8 T cells and macrophages that were similar to frequencies observed in naïve mice during a challenge (10^7^ IrrPb Challenged, black and hatched bars). The mean difference in brain-sequestered CD8 T cells from 10^7^ IrrPb challenged vs. naïve challenged mice was −.9 (−2.8, 1.0)%, p = .12, Two Way ANOVA. **C**) Graph of the number of cells analyzed for each group. The absolute numbers of total cells and CD8 T cells analyzed were not significantly different between the 10^7^ IrrPb immunized and naïve groups, p>.5, Two Way ANOVA. **D**) Brain-sequestered CD8 T cells from both immunized and naïve mice largely had a CD44+ CD62L− effector phenotype (hatched bars), and **E**) approximately 55% of these effector cells were also CD69+ in both Naïve mice and 10^7^ IrrPb immunized mice after challenge.

We next examined the immune cell subtypes in the brains of mice after a single immunization with 10^7^ IrrPb for correlations with the protection from ECM (Figure 6). As reported previously, CD8 T cells and macrophages were prominent in the brain vasculature of *Pb-A* infected naïve mice on day 6 (Figure 6A, and 6B, Naïve Challenged, solid black and hatched bars, 4.2+/−2.1% and 2.6+/−0.7% of isolated cells, respectively). Microglia, NK cells, and NKT cells were detected at low frequencies in these experiments, and significant differences were not detected among any of the sample groups (Figure 6B). The number of total cells and specific cell types analyzed in each group are shown in Figure 6C.

Surprisingly, CD8 T cells and macrophages were also detected in the brains of mice immunized with 10^7^ IrrPb on day 6 after challenge, and their frequencies were not significantly different from those observed in naïve mice during infection (Figure 6B, 10^7^ IrrPb Challenged, solid black and hatched bars, 3.3+/−0.2% and 2.0+/−0.6% of isolated cells, respectively). In agreement with previous reports in the *Pb-A* model, the brain-sequestered CD8 T cells from infected naïve and immunized mice largely had a CD44+ CD62L− effector phenotype (Figure 6D, hatched bars), and approximately 55% of these effector cells were also positive for the early activation marker CD69+ (Figure 6E) consistent with active, tissue invading cells. This indicated that immunization with 10^7^ IrrPb protected mice from ECM after a virulent *Pb-A* challenge without significantly reducing the infiltration of CD 8 T cells into the brain vasculature.

## Discussion

Some of the earliest malaria vaccine studies in experimental models were based on the use of irradiation-attenuated whole malaria parasites [24]. However, due to the perceived difficulties related to their safety and large scale production, the whole parasite approach was not considered to be a practical vaccine strategy. In recent years, the limited success of sub-unit based vaccines in clinical studies has reignited the interest in whole parasite based malaria vaccines [11]. Notwithstanding their potential value as candidate vaccines, studies with radiation-attenuated parasites offer an excellent opportunity to explore host pathogenesis and to examine the immune mechanisms induced by protective vaccines against malaria.

In the current study, a single, non-adjuvanted immunization with 10^7^ irradiated blood-stage parasites protected CD1 mice from parasitemia and severe disease, while up to two immunizations with 10^3^ irradiated parasites were not protective. This anti-parasite immunity is consistent with previous studies of irradiated blood-stage malaria parasites that showed protection from parasitemia and severe malaria anemia in different model systems [12], [13], [24], [25], [26]. The protection in the previous studies was achieved without adjuvants, and it also required the intravenous delivery of large numbers of blood-stage parasites.

To our knowledge, the current study is the first to also demonstrate the effectiveness of irradiated blood-stage parasites for protection against ECM, the most pathogenic consequence of malaria infection. A single immunization with 10^7^ irradiated parasites protected against ECM, while two immunizations of 10^3^ irradiated parasites did not protect. However, in contrast to immunized CD1 mice, the immunized C57BL/6 mice that had acquired anti-disease immunity against ECM did not acquire anti-parasite immunity. Similar observations have been noted in other experimental studies [23], [27], suggesting that anti-disease immunity and anti-parasite immunity act through distinct mechanisms. This notion is further supported by the observations of distinct immune mechanisms that confer immunity against severe malaria and parasitemia in adults living in endemic areas [28], [29].

The mechanism of protection in this study is not known, and thus it is not clear what relative roles humoral and cellular responses may have played for this protection. The protective immunization against ECM in this study was associated with a parasite-specific antibody response that increased following a boost during a challenge infection. However, whether these antibodies played any role against protection from ECM (C57Bl/6 mice) or parasitemia (CD1 mice) is not clear. In previous studies, antibody responses have been shown to be important to some whole parasite vaccines [14], [16], and it has been proposed that part of their protective effects may come from an increased clearance of opsonized parasites [30]. In the current study, the immunized ECM-protected mice did not have significantly lower blood parasitemia levels compared to non-protected mice, suggesting that bulk parasite clearance is unlikely to fully explain protection. However, the spleen sizes of protected mice increased 2-fold during a virulent infection, and since this can be indicative of increased parasite clearance and/or immune cell recruitment, further experiments will be necessary to address this question in detail. The ECM-protective immunization was also associated with a reduced parasite-specific IFN-γ response in the spleen during a virulent infection. In mice that have a Th1 bias such as C57BL/6, splenic IFN-γ production during a blood-stage *Pb-A* infection is thought to promote inflammatory responses that contribute to ECM pathology [31]. In contrast, the non-protective immunization in this study did not stimulate anti-parasite antibodies and it only modestly reduced parasite-specific IFN-γ expression during an infection, indicating that it was less effective at stimulating both antibody and cellular responses.

While elevated levels of splenic IFN-γ during the fulminant phase of Pb-A infection in mice are associated with susceptibility to ECM, a very early IFN-γ response in the spleen during infection has been associated with resistance to ECM [32]. In a previous study, it was proposed that CD 8+ T cells were a source of the early splenic IFN-γ responses during *Pb-A* infection, and the authors provided evidence that NK cells, NKT cells, and γδ T cells did not significantly contribute to this IFN-γ signal. In the current study, the unchallenged, IrrPb immunized mice produced detectable levels of splenic IFN-γ when stimulated in vitro with *Pb-A* antigen. While the source of this splenic IFN-γ produced upon immunization with IrrPb was not studied, we cannot discount the possibility that antigenically primed CD 4+ T cells or some other splenic cells were responsible for the IFN-γ production through an immunological feedback mechanism, since ECM-protected IrrPb immunized mice had lower splenic IFN-γ levels after *Pb-A* challenge (Figure 5).

Recently, it has been proposed that immunization with subpatent doses of viable blood-stage parasites that have been genetically attenuated or curtailed by drug cure can provide protection from a virulent challenge [16], [18], [33]. These are intriguing findings, and may in part be linked to the degree to which the attenuated parasites are able to replicate and persist to stimulate the immune system without causing severe disease symptoms. In the current study, even the largest inoculums of irradiated parasites fell below the limit of blood-smear detection within a few days, suggesting that these parasites were capable of little or no replication. This level of attenuation was necessary because it appeared that replication competent blood-stage *Pb-A* also remained virulent to produce fulminant infections and severe disease. In another study, genetically attenuated, blood-stage *Pb-A* parasites that retained an ability to replicate carried a very high risk of severe disease themselves, although mice that survived the initial vaccination developed robust immunity against both parasites and disease during subsequent challenge infections [14]. In the current study, low numbers of the non-replicating parasites failed to induce anti-parasite or anti-disease immunity.

Although immunization with a high dose of irradiated *Pb-A* protected mice against ECM in this study, the protected mice still accumulated CD8 T cells in their brain vasculature similar to ECM-susceptible mice. Previous studies have shown that brain sequestered CD8 T cells are necessary but not sufficient for ECM in the *Pb-A* model [34], [35], [36], [37]. Similar to this study, perforin-deficient mice that were resistant to ECM still accumulated activated effector CD8 T cells (CD44+, CD62L−, CD69+) in their brains [35]. Further experimentation will be needed to explore the mechanism of ECM resistance induced by irradiated blood stage parasites.

In summary, inline with our results in the *P. berghei* model, it is possible that a single inoculation with a high dose of replication-deficient *P. falciparum* parasites might protect children in endemic areas from parasite burden and/or cerebral malaria. In either scenario, such a vaccine might lower the prospect of death from severe malaria during their vulnerable early years and thus provide them with the opportunity to develop clinical immunity after continued parasite exposure.
